# Control of the dynamics of a boiling vapour bubble using pressure-modulated high intensity focused ultrasound without the shock scattering effect: A first proof-of-concept study

**DOI:** 10.1016/j.ultsonch.2021.105699

**Published:** 2021-07-31

**Authors:** Ki Joo Pahk

**Affiliations:** a Center for Bionics, Biomedical Research Institute, Korea Institute of Science and Technology (KIST), Seoul 02792, Republic of Korea; b Division of Bio-Medical Science & Technology, KIST School, University of Science and Technology (UST), Seoul 02792, Republic of Korea

**Keywords:** High intensity focused ultrasound, Boiling histotripsy, Pressure-modulated shockwave histotripsy, Shock scattering, Acoustic cavitation, Bubble coalescence

## Abstract

Boiling histotripsy is a promising High-Intensity Focused Ultrasound (HIFU) technique that can be used to induce mechanical tissue fractionation at the HIFU focus *via* cavitation. Two different types of cavitation produced during boiling histotripsy exposure can contribute towards mechanical tissue destruction: (1) a boiling vapour bubble at the HIFU focus and (2) cavitation clouds in between the boiling bubble and the HIFU source. Control of the extent and degree of mechanical damage produced by boiling histotripsy is necessary when treating a solid tumour adjacent to normal tissue or major blood vessels. This is, however, difficult to achieve with boiling histotripsy due to the stochastic formation of the shock scattering-induced inertial cavitation clouds. In the present study, a new histotripsy method termed pressure-modulated shockwave histotripsy is proposed as an alternative to or in addition to boiling histotripsy without inducing the shock scattering effect. The proposed concept is (a) to generate a boiling vapour bubble *via* localised shockwave heating and (b) subsequently control its extent and lifetime through manipulating peak pressure magnitudes and a HIFU pulse length. To demonstrate the feasibility of the proposed method, bubble dynamics induced at the HIFU focus in an optically transparent liver tissue phantom were investigated using a high speed camera and a passive cavitation detection systems under a single 10, 50 or 100 ms-long 2, 3.5 or 5 MHz pressure-modulated HIFU pulse with varying peak positive and negative pressure amplitudes from 5 to 89 MPa and −3.7 to −14.6 MPa at the focus. Furthermore, a numerical simulation of 2D nonlinear wave propagation with the presence of a boiling bubble at the focus of a HIFU field was conducted by numerically solving the generalised Westervelt equation. The high speed camera experimental results showed that, with the proposed pressure-modulated shockwave histotripsy, boiling bubbles generated by shockwave heating merged together, forming a larger bubble (of the order of a few hundred micron) at the HIFU focus. This coalesced boiling bubble then persisted and maintained within the HIFU focal zone until the end of the exposure (10, 50, or 100 ms). Furthermore, and most importantly, no violent cavitation clouds which typically appear in boiling histotripsy occurred during the proposed histotripsy excitation (i.e. no shock scattering effect). This was likely because that the peak negative pressure magnitude of the backscattered acoustic field by the boiling bubble was below the cavitation cloud intrinsic threshold. The size of the coalesced boiling bubble gradually increased with the peak pressure magnitudes. In addition, with the proposed method, an oval shaped lesion with a length of 0.6 mm and a width of 0.1 mm appeared at the HIFU focus in the tissue phantom, whereas a larger lesion in the form of a tadpole (length: 2.7 mm, width: 0.3 mm) was produced by boiling histotripsy. Taken together, these results suggest that the proposed pressure-modulated shockwave histotripsy could potentially be used to induce a more spatially localised tissue destruction with a desired degree of mechanical damage through controlling the size and lifetime of a boiling bubble without the shock scattering effect.

## Highlights

• A new histotripsy concept is proposed and demonstrated.
• The extent and lifetime of a boiling bubble can be controlled.
• Boiling bubbles formed by localised shockwave heating can merge together.
• The coalesced boiling bubble maintains at the focus until the end of the exposure.
• No shock scattering-induced cavitation clouds are produced with the proposed method.

## 1 Introduction

Boiling histotripsy is a promising High Intensity Focused Ultrasound (HIFU) technique which uses a number of millisecond long HIFU pulses with high peak positive *P_+_* and negative *P_−_* pressures at the HIFU focus, resulting in mechanical tissue fractionation *via* acoustic cavitation [1], [2], [3]. Numerous previous studies have shown that boiling histotripsy can be used to destroy different types of soft tissues (e.g., heart, kidney, brain, prostate and liver) without causing any significant thermal damage [2], [3], [4], [5], [6], [7], [8], [9], [10]. The overall shape of a boiling histotripsy lesion is tadpole like, consisting of a head and a tail with the head located towards the HIFU transducer [11]. In boiling histotripsy, shockwaves formed at the HIFU focus in soft tissue due to tissue nonlinear effect can initially raise tissue temperature to boiling in a few milliseconds (i.e., shock wave heating) [1], [2]. A boiling vapour bubble is subsequently generated at the HIFU focus. Further interactions of this boiling bubble with incoming incident shockwaves cause mechanical tissue damage [3], [4], [6]. Once a boiling bubble interacts with shockwaves, the bubble can expand to around a millimetre through rectified growth behaviour [10], [12]. This rectification motion, which is due to the combination of the asymmetry in a shockwave and water vapour transport [12], [13], has been experimentally observed in tissue phantom with a high speed camera [10]. Shear stresses produced around this growing boiling bubble can mechanically fractionate surrounding soft tissue [10]. Following this rectified growth, the shock scattering effect [4], [14], which is the formation of additional bubble nucleation sites by the constructive interference of the backscattered shockwave by a bubble with incoming incident shockwaves, then takes place, which can generate an inertial cavitation cluster (i.e., violent bubble collapses) in front of the boiling bubble, progressing toward the HIFU transducer [15]. Along with this cavitation clouds, additional boiling bubbles can form within the HIFU focal region through incoming wave diffraction [15]. A number of boiling bubbles generated within a localised shockwave heated zone together with cavitation clouds produced between the boiling bubble and the HIFU source are, respectively, responsible for the formations of the tail and the head of a boiling histotripsy lesion [15], [16].

Precise control of the extent and degree of mechanical damage induced by boiling histotripsy is necessary, especially when treating a solid tumour adjacent to normal tissue or major blood vessels. This is, however, difficult to achieve because of the generation of the shock scattering induced violent cavitation clouds during boiling histotripsy exposure. This cavitation cluster formation is highly dependent upon the pressure magnitude of a backscattered acoustic field by a boiling bubble [14] and the location and number of pre-existing bubble nuclei within soft tissue [2], [17]. These make it difficult to accurately predict and control the spatio-temporal activity of cavitation clouds formed under a given boiling histotripsy exposure condition.

**Fig. 1:**
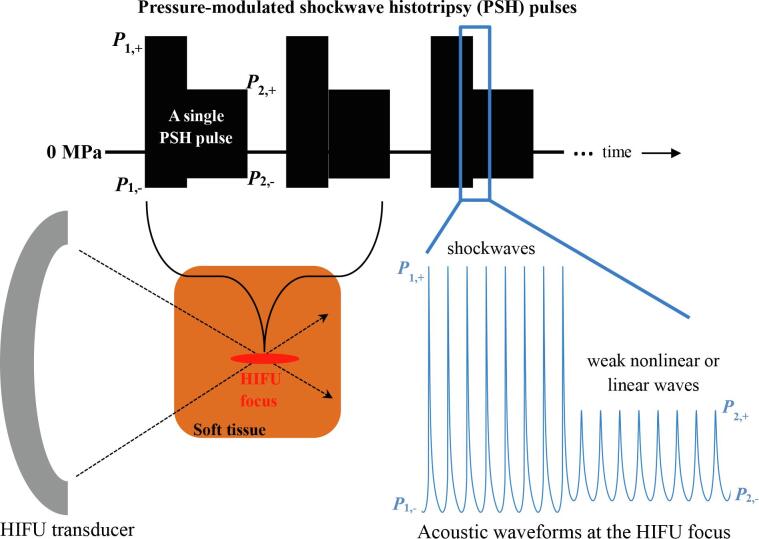
Illustration of the proposed pressure-modulated shockwave histotripsy pulsing protocol to control the extent and lifetime of a boiling bubble without inducing the shock scattering effect. *P*_1,+_ and *P*_1,−_ are the peak positive and negative pressures in shockwaves at the HIFU focus. *P*_2,+_ and *P*_2,−_ are those in the subsequent HIFU waves within a single pressure-modulated shockwave histotripsy pulse.

It has been reported that the extent of a lesion and degree of mechanical damage induced by boiling histotripsy depend upon the size of a boiling bubble [16] and the number of boiling histotripsy pulses applied [2], [6], respectively. The size and lifetime of a bubble at the HIFU focus would therefore be two important elements when controlling the extent and degree of mechanical damage generated: the smaller the bubble size, the smaller the extent of a histotripsy lesion. The longer the bubble lifetime, the higher the degree of mechanical damage produced, and vice versa. With these contexts, in the present study, a new histotripsy method termed pressure-modulated shockwave histotripsy is proposed to control the size and lifetime of a boiling bubble without inducing the shock scattering effect. The proposed concept is to use a pressure-modulated HIFU pulse, where (a) shockwaves with high acoustic pressure amplitudes (*P*_1,+_ and *P*_1,−_) are initially used to produce a boiling bubble and (b) subsequent weakly distorted nonlinear or linear waves with lower pressure amplitudes (*P*_2,+_ and *P*_2,−_) are applied to control the bubble dynamics (See Fig. 1). Specifically, shockwave heating would initially increase tissue temperature to boiling creating a boiling vapour bubble at the HIFU focus. The size and lifetime of this boiling bubble would then be controlled by the peak pressure magnitudes (*P*_2,+_ and *P*_2,−_) and the length of the subsequent HIFU waves used in the pressure-modulated HIFU pulse. With the proposed strategy, no shock scattering effect would likely occur after the formation of a boiling bubble because the backscattered peak negative pressure magnitude by the boiling bubble would be kept below the cavitation cloud intrinsic threshold of –28 MPa, which is the reported threshold for negative pressure value at which violent cavitation clouds are almost certain to appear in soft tissue [17], [18]. To demonstrate the feasibility of the proposed idea, the dynamics of a boiling bubble induced at the HIFU focus in an optically transparent tissue phantom during the course of a pressure-modulated HIFU excitation are investigated using a high speed camera and a passive cavitation detection (PCD) systems. A numerical simulation on nonlinear wave propagation with a bubble at the HIFU focus is also performed to capture basic features of the backscattered acoustic fields.

## 2 Methods

### 2.1 High speed camera experimental set up

**Fig. 2:**
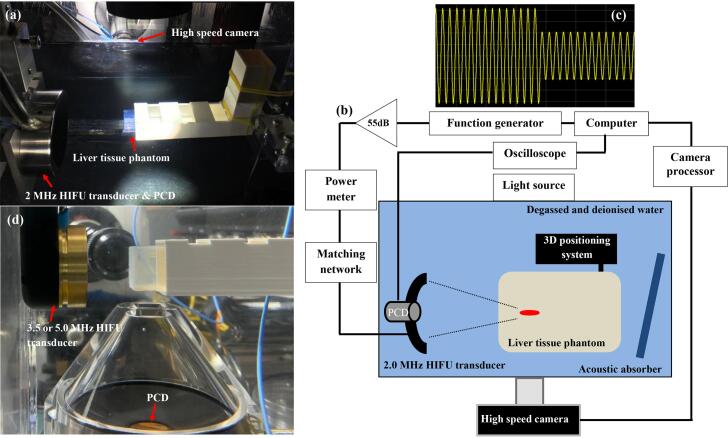
(a) A photograph and (b) a schematic diagram showing the HIFU experimental set up with the 2 MHz HIFU transducer. (c) shows an example of amplitude modulated sinusoidal signals that were employed to drive the HIFU transducer. (d) A photograph of the experimental set up used with the 3.5 or the 5.0 MHz HIFU transducer.

A schematic diagram of the HIFU experimental set up used in the present study is shown in Fig. 2. The HIFU experiments performed with an optically transparent liver tissue phantom were carried out in an acrylic water bath filled with degassed and de-ionised water at 20 °C. Three different single element bowl-shaped HIFU transducers operating at 2.0, 3.5 or 5.0 MHz were used, which were driven by a function generator (33600A, Agilent, CA, USA) and a RF power amplifier (1040L, ENI, NY, USA). Characteristics of the HIFU transducers are provided in Table 1. A computer with waveform generation software (Agilent Waveform Builder, Agilent) was used for driving the function generator with the desired pressure-modulated HIFU pulsing protocol (Fig. 2c). A power meter (Sonic Concepts 24B, Bothell, WA, USA) was connected between the HIFU transducer and the power amplifier in order to measure the electrical power *P*_elect_ supplied to the transducer at a given driving condition. The same recipe for making an optically transparent liver tissue phantom (see Supplementary Table 1) used in previous boiling histotripsy studies [2], [10], [15], [16], [19] was employed in the present study to prepare a cuboid liver tissue phantom (10 cm × 10 cm × 2 cm). This liver tissue phantom has similar acoustic and thermal properties to those of normal liver tissue with elasticity of 4.85 kPa [10], [20]. A total number of 21 tissue phantoms (*n* = 21) was used in this study. The distances between the surface of the HIFU transducers and the phantom were respectively 58.2 mm, 30 mm and 30 mm for the 2.0, 3.5 and 5.0 MHz transducers. An acoustic absorber (AptFlex F28, Precision Acoustics Ltd, Dorset, UK) was also placed on the opposite end to the HIFU transducer in the water bath to minimise ultrasonic reflections. Most of the analyses performed in the present study were based upon results obtained with the 2 MHz HIFU source. In boiling histotripsy, one of the most frequently used frequencies is 2 MHz [1], [2], [5], [6], [9], [10], [15], [16], [21], [37], and the mechanisms of boiling histotripsy have been extensively investigated with a high speed camera under 2 MHz sonication [1], [2], [9], [10], [15], [16].

**Table 1:** Characteristics of the HIFU transducers used in the present study.

Model & Manufacturer	Frequency	Aperture size	Radius of curvature	Central hole size	Axial full width half maximum (FWHM) pressure dimension in water	Lateral FWHM pressure dimension in water
H148/Sonic Concepts, Bothell, WA, USA	2 MHz	64 mm	63.2 mm	22.6 mm	*7.25 mm	*0.89 mm
SU-107/Sonic Concepts	3.5 MHz	33 mm	35 mm	No central hole	#3.5 mm	#0.45 mm
SU-108/Sonic Concepts	5.0 MHz	33 mm	35 mm		#2.65 mm	#0.32 mm

* Calibrated and measured in [21].

# Provided by the manufacturer.

During the experiments, HIFU-induced bubble dynamics at the HIFU focus in the liver tissue phantom were captured using a high speed camera (Phantom V2512, Vision Research, New Jersey, USA) with a 9× Navitar lens (Navitar, Rochester, NY, USA). The camera was operated at 0.11 M frames per second (fps), a shutter speed of 4.5 μs and a pixel resolution of 512 by 320 (i.e., 6.2 μm per a pixel). All high speed camera experiments were backlit with an illuminating system (Cyclops I-120 W, KOMI, Gyonggi-do, Republic of Korea). The camera and the function generator were triggered at the same time using a built-in camera software (Phantom Camera Control PCC, Vision Research). All captured high speed images were post-processed with the PCC software to investigate the changes in the sizes of a boiling bubble and a lesion by counting the pixels. In addition to optical observations of bubble dynamics, acoustic emissions emitted at the HIFU focus in the liver tissue phantom during the HIFU exposure were also recorded using a 10-MHz focused PCD transducer (20 mm in diameter and 64 mm in geometric focal length, bandwidth of 10 kHz to 20 MHz, Y107, Sonic Concepts). This PCD transducer was inserted into the central hole of the 2.0 MHz HIFU transducer and was connected to a digital oscilloscope (LeCroy HDO 6054, Berkshire, UK). For the experiments performed with the 3.5 MHz or the 5.0 MHz transducer, the PCD transducer was placed bottom surface of the water bath facing upward, as shown in Fig. 2(d). A sampling frequency of 1 GHz was used.

### 2.2 Pressure-modulated shockwave histotripsy exposure conditions

In the high speed camera experiment, a single 10 ms-long pressure-modulated HIFU pulse was employed to demonstrate the proposed pressure-modulated shockwave histotripsy. The HIFU exposure conditions used were determined according to the following criteria:

• Boiling appears in times much shorter than the characteristic heat diffusion time scale of 19 ms in the liver tissue phantom such that thermal diffusion effect can be neglected [1].
• Initial acoustic peak positive *P*_1,+_ and negative *P*_1,−_ pressures at the HIFU focus are high enough to increase tissue phantom temperature to boiling temperature within 5 ms. Previous boiling histotripsy studies [2], [10], [16] have used 1 to 3 MHz HIFU waves with *P*_+_ of 67 to 85.4 MPa and *P_−_* of 9 to 15.6 MPa to generate a boiling vapour bubble at the HIFU focus in the liver tissue phantom within 5 ms. Here, a shockwave is defined as a strongly distorted nonlinear wave with a rapid rise time of < 50 ns, *P*_+_ ≥ 35 MPa and *P_−_* of about 10 MPa [22].
• After the formation of a boiling bubble at the HIFU focus, a few more acoustic cycles with *P*_1,+_ and *P*_1,−_ are applied in order for the bubble to undergo rectified growth.
• Lastly, lower peak positive *P*_2,+_ and negative *P*_2,−_ pressure values than *P*_1,+_ and *P*_1,−_ are subsequently employed to avoid or minimise the shock scattering effect.

Acoustic peak pressure values at the HIFU focus in the tissue phantom (*P*_1,+_, *P*_1,−_ and *P*_2,+_, *P*_2,−_) were obtained by numerically solving the Khokhlov-Zabolotskaya-Kuznetsov (KZK) nonlinear wave equation and the corresponding temperature rise was computed using the Bio-heat Transfer equation for a set of input parameters [10], [15]. It has been reported that a numerical modelling of wave propagation using the KZK equation provides better accuracy in predicting nonlinear waveforms, particularly a shockwave front than experimental measurements because of a limited bandwidth of a hydrophone used for measuring strong nonlinear HIFU fields [23]. The HIFU Simulator v1.2 [24] was employed for simulating acoustic and temperature fields produced in the tissue phantom. In the KZK simulations, for simplicity, the effects of the changes in temperature-dependent acoustic properties on wave propagation were not accounted for [15]. Acoustic and thermal properties of the liver tissue phantom used in the simulations are given in Supplementary Table 2. Here, boiling is assumed to be at 100 °C [2], [10], [23].

On the basis of the criteria of potential pressure-modulated shockwave histotripsy exposure conditions outlined above, a various range of HIFU exposure conditions with driving frequencies of 2.0, 3.5 and 5.0 MHz, and *P_+_* from 5 MPa to 89 MPa and *P_−_* from −3.7 to −14.6 MPa was tested in the present study, which are shown in Table 2. For the 2 MHz HIFU transducer, for instance, 2 MHz HIFU waves with *P*_1,+_ of 89.1 MPa and *P*_1,−_ of −14.6 MPa were initially applied during the first 4 ms of a 10 ms long pressure modulated HIFU pulse. With *P*_1,+_ of 89.1 MPa and *P*_1,−_ of −14.6 MPa, the time-to-boil was numerically predicted to be 3.98 ms. Then (a) *P*_2,+_ of 29.9; *P*_2,−_ of −9.6 MPa, (b) *P*_2,+_ of 13.2; *P*_2,−_ of −6.8 MPa, or (c) *P*_2,+_ of 5.0; *P*_2,−_ of −3.7 MPa were employed during the subsequent 6 ms of the exposure (i.e., a time period between 4 ms and 10 ms).

**Table 2:** Pressure-modulated shockwave histotripsy exposure conditions used in the present study.

A single 10 ms-long pressure-modulated shockwave histotripsy pulse									
		1st HIFU waves				2nd HIFU waves			
Frequency (MHz)	*P*_elect_ (W)	*P*_+,1_ (MPa)	*P_−_*_,1_ (MPa)	*Simulated time to reach 100 °C (ms)	Exposure time (ms)	*P*_elect_ (W)	*P*_+,2_ (MPa)	*P_−_*_,2_ (MPa)	Exposure time (ms)
2	200	89.1	−14.6	3.98 (*T*_0_ = 20 °C)	4	55	29.9	−9.6	6
						22	13.2	−6.8	6
						4	5.0	−3.7	6
3.5	55	72.4	−13.8	4.48 (*T*_0_ = 20 °C)	5	19	32.1	−9.6	5
						10	17.6	−7.7	5
						4	8.8	−5.4	5
5.0	28	69.2	−12.5	3.68 (*T*_0_ = 10 °C)	5	9	29.2	−8.6	5
						4	13.9	−6.4	5

*T*_0_ = initial temperature of tissue phantom.

### 2.3 Numerical modelling of scattered acoustic fields around a bubble

**Fig. 3:**
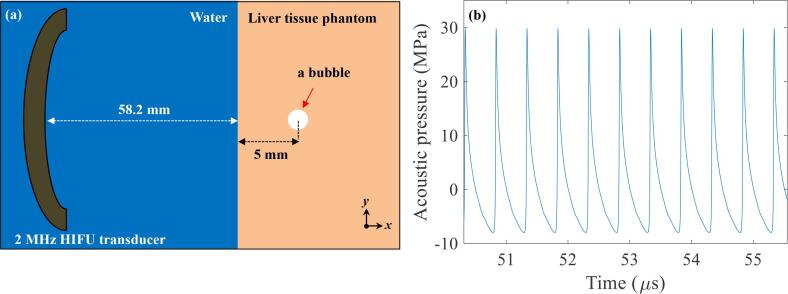
(a) A geometrical model used in the k-Wave simulations. (b) Computed 1D waveforms (*P_+_* = 28 MPa, *P_−_* = – 8 MPa) at 63.2 mm in the HIFU axial direction in the absence of a bubble at the focus.

A 2D numerical simulation under a given *P*_2,+_ and *P*_2,−_ was performed to capture essential features of 2 MHz nonlinear acoustic fields produced around a vapour bubble in the tissue phantom using the open source k-Wave v1.2 MATLAB toolbox [25]. k-Wave, which numerically solves the generalised Westervelt equation that accounts for heterogeneities in the ambient mass density, material nonlinearity (second-order nonlinearity) and power law absorption and dispersion, has been successfully used to study the interaction of shockwaves (*P_+_* of 51.2 MPa and *P_−_* of −9.8 MPa) with a boiling bubble in boiling histotripsy [14]. The same geometrical 2D model developed and implemented in [14] was employed in the present study (Fig. 3a). The total grid size employed was 2^14^ × 2^14^ points with a computational domain size of 75.7 mm × 75.7 mm. In addition, 160 points per wavelength at 2 MHz and a CFL number of 0.05 with a temporal step size of 0.15 ns and a grid spacing of 4.63 μm in the axial and lateral directions were used. All simulations were carried out on a desktop PC with 3.6 GHz CPU (i9-9900 K), 64 GB of RAM and NVIDIA GeForce RTX 8000 (48 GB) GPU. Each simulation took four days to complete.

Since it has been previously pointed out that the peak positive phase of an incident shockwave has a great impact on the peak negative pressure magnitude of backscattered acoustic fields by a bubble [14], [17], the pressure amplitude of an input sinusoidal signal used in the k-Wave simulations performed was varied until the peak positive pressure value in the absence of a bubble at the HIFU focus became similar to *P*_2,+_ of 29.9 MPa (Fig. 3b) (within a difference of 10% in the pressure magnitude). The size of a bubble used in the simulation was obtained from high speed camera experimental results.

## 3 Results

### 3.1 During the conventional boiling histotripsy exposure – 2 MHz

**Fig. 4:**
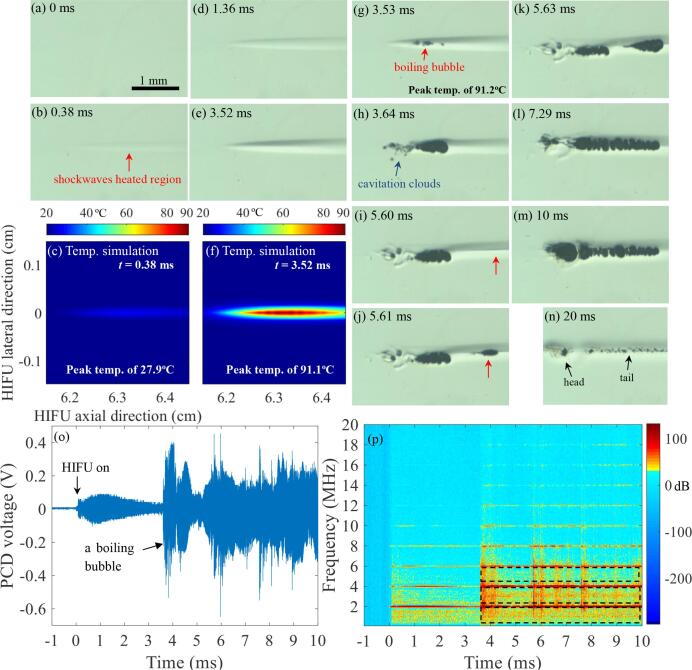
A series of high speed images of the bubble dynamics induced at the HIFU focus in the tissue phantom during a single 10 ms-long 2 MHz boiling histotripsy pulse with *P_+_* of 89.1 MPa and *P_−_* of –14.6 MPa. Images were obtained at a 0.11 M fps. The times at (a) 0 ms and (m) 10 ms correspond to the start and the end of the boiling histotripsy exposure. An image (n) was captured 10 ms after the HIFU ceased at *t* = 10 ms. (c) and (f) show the computed 2D temperature contour plots at *t* = 0.38 ms and *t* = 3.52 ms, respectively. (o) PCD voltage versus time plot. (p) the corresponding spectrogram of (o). The HIFU beam propagates from left to right.

Bubble dynamics resulting from boiling histotripsy exposure were initially captured here for comparison purpose with the proposed pressure-modulated shockwave histotripsy. Fig. 4 shows a series of high speed images obtained during a single 10 ms-long 2 MHz boiling histotripsy pulse in the tissue gel phantom with *P_+_* of 89.1 MPa and *P_−_* of 14.6 MPa. Similar to the previous boiling histotripsy studies [2], [10], [15], [16], localised shockwave heating in the form of a dark elliptical shape occurs within the HIFU focal volume at *t* = 0.38 ms (Fig. 4b), which corresponds well to the computed 2D temperature contour plot depicted in Fig. 4c. This localised heated region enlarges over time (Fig. 4b–f). With *P_+_* = 89.1 MPa and *P_−_* = 14.6 MPa, the time to reach the boiling temperature of 100 °C was predicted to be 3.98 ms (Table 2). At *t* = 3.53 ms (simulated corresponding peak temperature of 91.2 °C), boiling bubbles with diameters ranging from 90 μm to 93 μm are observed within the localised heated zone (indicated by the red arrow in Fig. 4g). These bubbles then grow to around 260 μm over 0.11 ms (Fig. 4h) (i.e., rectified growth behaviour). It has been shown that boiling bubble nucleation in boiling histotripsy can happen below 100 °C since millisecond HIFU heat deposition can lower bubble nucleation pressure thresholds [26]. Following the rectified growth event, cavitation clouds can be seen at *t* = 3.64 ms in front of the boiling bubble towards the HIFU transducer (indicated by the blue arrow in Fig. 4h). This cavitation cluster formation in boiling histotripsy is most likely to be due to the shock scattering [14] and the pre-focal heating [14] which reduces the cavitation intrinsic threshold [27]. Vlaisavljevich et al [27] have reported that the intrinsic threshold in water-based tissue phantom decreases from −29.8 MPa at 10 °C to −14.9 MPa at 90 °C.

During the experiments, the time to boiling for the single 2 MHz HIFU pulse with *P_+_* of 89.1 MPa and *P_−_* of 14.6 MPa in the tissue phantom was 3.61 ms ± 0.11 ms (mean ± standard deviation SD with *n* = 8) with differences of 0.37 ms between the high speed camera observations and the temperature simulation.

At *t* = 5.6 ms, a secondary localised heated region (indicated by the arrow in Fig. 4i) at ~0.87 mm away from the primary boiling bubble further along the HIFU axial axis appears, and then secondary boiling bubbles subsequently form (indicated by the red arrow in Fig. 4j). These secondary generations of the heated region and boiling bubbles are likely to be due to the shielding effect caused by the cavitation cluster together with the primary boiling bubble as well as the diffraction of the incoming HIFU waves [15]. After the boiling histotripsy exposure, mechanical damage in the form of a tadpole shape consisting of a head and a tail is clearly visible in the tissue phantom, as shown in Fig. 4(n). The length (along the direction of wave propagation) and width (the lateral direction) of the mechanical damage occurred at the HIFU focus after the boiling histotripsy exposure are measured to be 2.72 mm and 0.25 mm, respectively. The extent of the mechanical damage produced is larger than that of the shockwave heated region depicted in Fig. 4(e).

In addition to the optical observations, indications of the formations of the primary boiling bubble and the cavitation cluster can be observed in the corresponding PCD results, which are shown in Fig. 4(o) and (p). An increase in the PCD voltage amplitude (Fig. 4o) and a sudden occurrence of higher order multiple harmonic components of the fundamental frequency (2 MHz) in the spectrogram (Fig. 4p) represent the formation of a boiling bubble [1], [13], whereas a significant appearance of broadband emissions is generally resulted from the presence of inertial cavitation clouds during boiling histotripsy exposure [10], [15]. This broadband noise is clearly noticed within the black dashed lines in the spectrogram after the generation of a boiling bubble at *t* = 3.53 ms, as shown in Fig. 4(p).

During the experiments, a boiling histotripsy lesion with the length of 2.63 ± 0.44 mm and width of 0.24 ± 0.02 mm (mean ± SD with *n* = 4) appears in the liver tissue phantom after the exposure.

### 3.2 During the proposed pressure-modulated shockwave histotripsy exposure – 2, 3.5, 5 MHz

#### 3.2.1 2 MHz

To demonstrate the feasibility of the proposed histotripsy in the present study, peak positive and negative pressure values at the HIFU focus during the first 4 ms of a 10 ms long-pressure modulated 2 MHz HIFU pulse were kept constant (i.e., *P*_1,+_ of 89.1 and *P*_1,−_ of –14.6 MPa), whilst those during the subsequent 6 ms (i.e., a time period between 4 ms and 10 ms) were varied as: (a) *P*_2_,_+_ of 29.9; *P*_2_,_−_ of –9.6 MPa, (b) *P*_2_,_+_ of 13.2; *P*_2_,_−_ of –6.8 MPa, or (c) *P*_2_,_+_ of 5.0; *P*_2_,_−_ of –3.7 MPa (see Table 2).

**Fig. 5:**
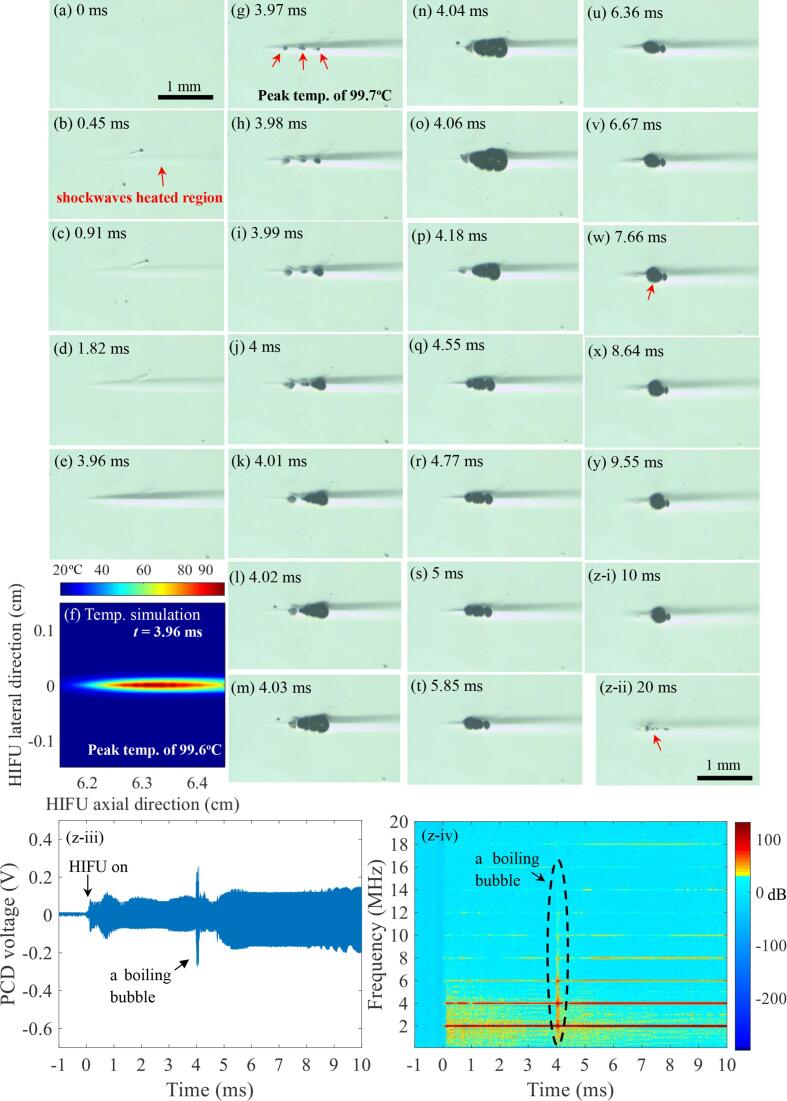
High speed camera images acquired at 0.11 Mfps during the proposed pressure-modulated shockwave histotripsy exposure. A single 10 ms-long pressure modulated 2 MHz HIFU pulse was employed with *P*_1,+_ of 89.1 and *P*_1,−_ of 14.6 MPa for the first 4 ms insonation and *P*_2_,_+_ of 29.9; *P*_2_,_−_ of –9.6 MPa during the subsequent 6 ms of the exposure. The times at (a) 0 ms and (z-i) 10 ms correspond to the start and the end of the pressure-modulated shockwave histotripsy exposure. An image (z-ii) was obtained 10 ms after the HIFU ceased at *t* = 10 ms. (f) represents the computed 2D temperature contour plot at *t* = 3.96 ms. The HIFU beam propagates from left to right. Peak pressures of 89.1 MPa and –14.6 MPa at the HIFU focus decreased to 29.9 MPa and –9.6 MPa at (j) *t* = 4 ms. (z-iii) PCD voltage versus time plot. (z-iv) the corresponding spectrogram of (z-iii). A movie showing the bubble dynamics captured over the 10 ms-long 2 MHz pressure-modulated shockwave histotripsy pulse with *P*_1_,_+_ of 89.1 and *P*_1_,_−_ of –14.6 MPa, and *P*_2_,_+_ of 29.9 and *P*_2_,_−_ of –9.6 MPa is available in Supplementary Video 1.

A number of high speed images obtained during the proposed pressure-modulated shockwave histotripsy excitation with *P*_2,+_ of 29.9 MPa and *P*_2,−_ of –9.6 MPa are depicted in Fig. 5. Localised shockwave heating appears at the HIFU focus (Fig. 5b–f) followed by the formation of boiling bubbles with diameters ranging from 49.2 μm to 63.2 μm at *t* = 3.97 ms (indicated by the red arrows in Fig. 5g). Indication of these boiling bubbles can also be seen in the corresponding PCD result (Fig. 5z-iii and z-iv). After when the peak positive and negative pressure magnitudes decrease from *P*_1_,_+_ = 89.1 MPa and *P*_1_,_−_ = –14.6 MPa to *P*_2_,_+_ = 29.9 MPa and *P*_2_,_−_ = –9.6 MPa at *t* = 4 ms (Fig. 5j), the boiling bubbles suddenly grow to about 316 ~ 422 μm (Fig. 5o) and then gradually shrink to 173 ~ 180 μm over time (Fig. 5p–s). Interestingly, after *t* = 5 ms, the boiling bubbles within the localised heated zone started to merge (i.e., bubble coalescence process commences) (Fig. 5t–v), forming a larger bubble with a diameter of 254 μm at *t* = 7.66 ms (indicated by the red arrow in Fig. 5w). This enlarged boiling bubble persists and remains at the HIFU focus until the HIFU pulse is switched off (Fig. 5z–i). The size of a boiling bubble at the end of the exposure (*t* = 10 ms) is measured to be 251.8 ± 69 μm (mean ± SD, *n* = 7) with *P*_2_,_+_ = 29.9 MPa and *P*_2_,_−_ = –9.6 MPa. In contrast to the results obtained with the boiling histotripsy exposure condition (Fig. 4), no secondary heated region (i.e., no secondary boiling bubble formation) as well as no significant cavitation clouds are optically detected with the absence of a significant appearance of broadband emissions in the corresponding PCD results (particularly after *t* = 4 ms in Fig. 5z-iii and z-iv).

Examining the phantom morphology after the pressure-modulated shockwave histotripsy exposure, residual mechanical damage of the tissue phantom is oval-shaped with a size of 0.61 mm (length) × 0.14 mm (width) and is visible at the HIFU focus (indicated by the arrow in Fig. 5z-ii). Indeed, the location of the mechanical damage induced corresponds well to that of the bubble at the end of the HIFU insonation. It is worth noting that the length and the width of the pressure-modulated shockwave histotripsy lesion (Fig. 5z-ii) are respectively measured to be about 4.5 and 1.8 times smaller than those of the boiling histotripsy lesion produced in the tissue phantom (Fig. 4n).

During the experiments, an oval shaped damaged lesion with the length of 0.55 ± 0.09 mm (along the direction of wave propagation) and width of 0.12 ± 0.03 mm (the lateral direction) is observed in the liver tissue phantom (mean ± SD with *n* = 7) after the pressure-modulated shockwave histotripsy exposure.

**Fig. 6:**
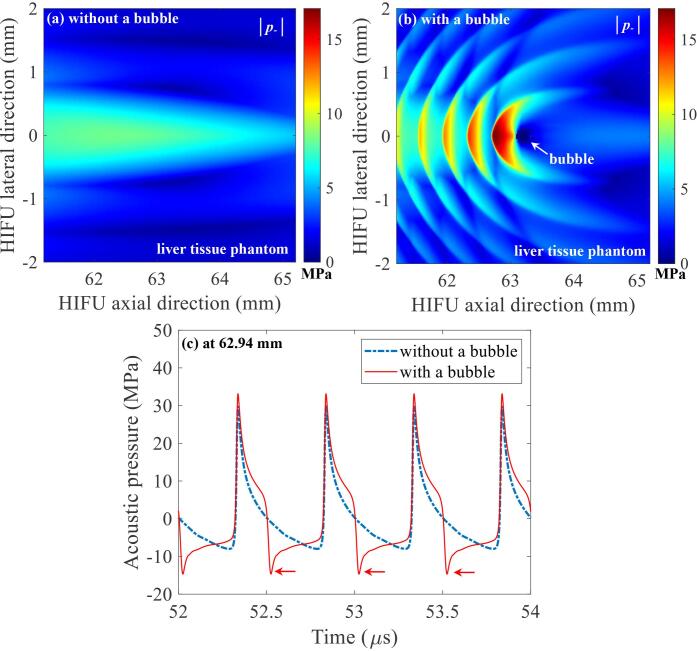
Computed 2D spatial distributions of negative pressure fields |*p*_-_| in the liver tissue phantom (a) without and (b) with a bubble at the HIFU focus. (c) is the simulated 1D waveforms at 62.94 mm in the HIFU axial direction. The 2 MHz HIFU beam propagates from left to right.

To investigate the changes of negative pressure fields after the formation of a boiling bubble during the proposed histotripsy exposure, a 2D nonlinear wave propagation was performed with the presence of a bubble at the HIFU focus. Fig. 6 shows the numerically simulated backscattered acoustic fields around a 217 μm-sized bubble at the HIFU focus in the tissue phantom. The bubble size was measured from Fig. 5(q). As can be seen, strong negative pressure fields between the HIFU source and the bubble are clearly observed with the highest peak negative pressure magnitude of –17.1 MPa appearing at 62.75 mm (in front of the bubble towards the HIFU source) along the HIFU axial direction (Fig. 6b). This value is about 2.1 times greater than that simulated without the bubble at the HIFU focus (Fig. 6a). It is, however, still below the cavitation cloud intrinsic threshold of –28 MPa. Due to the constructive and destructive interferences of the scattered acoustic field by the bubble with the incoming incident HIFU fields, the generation of localised peak negative pressures in the form of a layered structure separated by ~0.4 mm (about half of the wavelength at 2 MHz) can be observed in Fig. 6b.

**Fig. 7:**
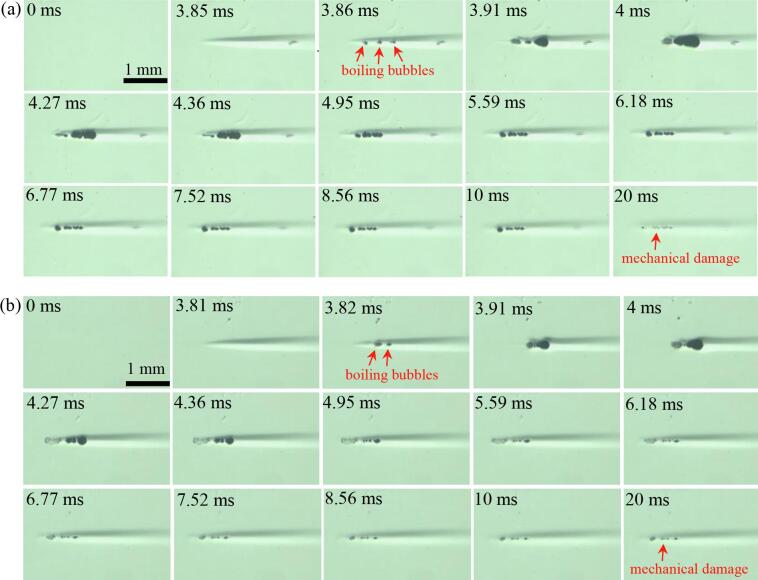
High speed images captured over a single 10 ms-long 2 MHz pressure-modulated shockwave histotripsy pulse with (a) *P*_1,_*_+_* of 86.1 MPa; *P*_1,−_ of –14.6 MPa and *P*_2,_*_+_* of 13.2 MPa; *P*_2,−_ of –6.8 MPa, and (b) *P*_1,_*_+_* of 86.1 MPa; *P*_1,−_ of –14.6 MPa and *P*_2,_*_+_* of 5.0 MPa; *P*_2,−_ of –3.7 MPa. The times at *t* = 0 ms and 10 ms correspond to the start and the end of the pressure-modulated shockwave histotripsy exposure. The 2 MHz HIFU beam propagates from left to right.

Fig. 7 depicts the effects of changes in the magnitudes of *P*_2,+_ and *P*_2,−_ on (a) the size of a boiling bubble at the end of the pressure-modulated shockwave histotripsy exposure and (b) the extent of mechanical damage produced in the tissue phantom. A positive relationship between the size of a boiling bubble and the extent of mechanical damage with increasing *P_2_*_,+_ and *P_2_*_,−_ is observed in Fig. 7. The maximum bubble size measured at the end of the exposure (i.e., at *t* = 10 ms) is measured to be 87.9 ± 15.6 μm (mean ± SD, *n* = 4) and 33.9 ± 3.7 μm (*n* = 4) with *P*_2,+_ = 13.2 MPa; *P*_2,−_ = –6.8 MPa, and *P*_2,+_ = 5 MPa; *P*_2,−_ = –3.7 MPa, respectively. Residual mechanical damage with a size of 0.51 ± 0.04 mm (length, *n* = 4) × 0.07 ± 0.01 mm (width, *n* = 4) appears at *P*_2,+_ = 13.2 MPa and *P*_2,−_ = –6.8 MPa, whereas a smaller lesion of a 0.47 ± 0.09 mm (length, *n* = 4) × 0.06 ± 0.004 mm (width, *n* = 4) is observed with *P*_2,+_ = 5 MPa and *P*_2,−_ = –3.7 MPa. This is summarised in Table 3.

**Table 3:** The effects of changes in pressure amplitudes on the bubble size and the lesion dimension under a given frequency.

Frequency [MHz]	Peak pressure amplitude [MPa]		Maximum bubble size [μm]	Final lesion size [mm]	
*f*_0_	*P*_2,+_	*P*_2,−_	Measured at *t* = 10 ms (mean ± SD)	Length along the direction of wave propagation	Width along the lateral direction of wave propagation
2	5.0	−3.7	33.9 ± 3.7 (*n* = 4)	0.47 ± 0.09	0.06 ± 0.004
	13.2	−6.8	87.9 ± 15.6 (*n* = 4)	0.51 ± 0.04	0.07 ± 0.01
	29.9	−9.6	251.8 ± 69 (*n* = 7)	0.55 ± 0.09	0.12 ± 0.03
	89.1*	−14.6*	476.2 ± 55 (*n* = 4)*	2.63 ± 0.44*	0.24 ± 0.02*

3.5	8.8	−5.4	51.5 ± 3.1 (*n* = 3)	0.30 ± 0.01	0.08 ± 0.01
	17.6	−7.7	114.7 ± 10.9 (*n* = 3)	0.33 ± 0.04	0.11 ± 0.01
	32.1	−9.6	224.2 ± 28.5 (*n* = 6)	0.41 ± 0.07	0.15 ± 0.02

5	13.9	−6.4	32.6 ± 2.9 (*n* = 3)	0.28 ± 0.03	0.05 ± 0.01
	29.2	−8.6	84.5 ± 11.9 (*n* = 5)	0.34 ± 0.02	0.06 ± 0.01

*In case of boiling histotripsy (i.e., no pressure modulation).

**Fig. 8:**
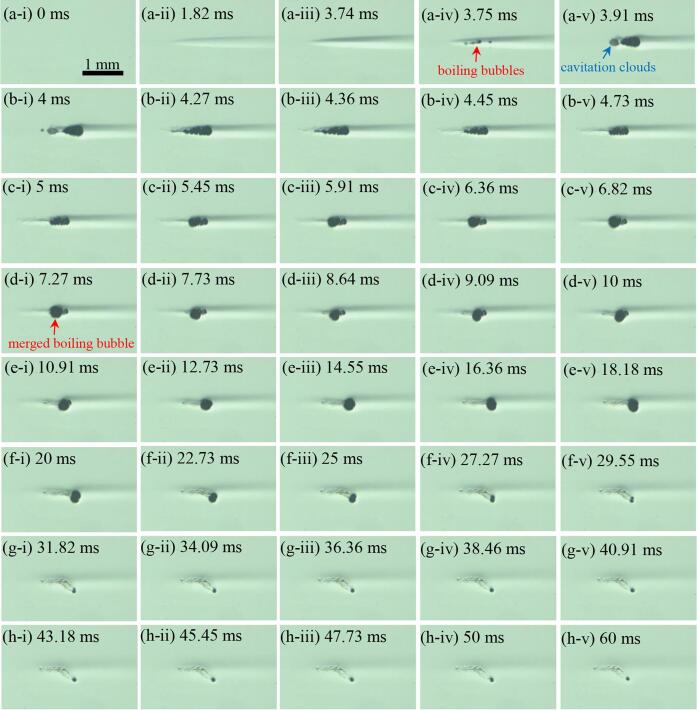
High speed images (a-i to h-v) taken during a 50 ms-long 2 MHz pressure-modulated shockwave histotripsy pulse with *P*_1,_*_+_* of 86.1 MPa; *P*_1,−_ of –14.6 MPa and *P*_2,_*_+_* of 29.9 MPa; *P*_2,−_ of –9.6 MPa. The peak pressures were changed at *t* = 4 ms. The times at (a-i) 0 ms and (h-iv) 50 ms correspond to the start and the end of the pressure-modulated shockwave histotripsy exposure. An image (h-v) was obtained 10 ms after the HIFU ceased at *t* = 50 ms. The 2 MHz HIFU beam propagates from left to right. A movie showing the bubble dynamics captured during the course of the 50 ms-long pressure-modulated shockwave histotripsy exposure with *P*_1_,_+_ = 89.1 MPa; *P*_1_,_−_ = –14.6 MPa and *P*_2_,_+_ = 29.9 MPa; *P*_2_,_−_ = –9.6 MPa is available in Supplementary Video 2.

**Supplementary figure 1:**
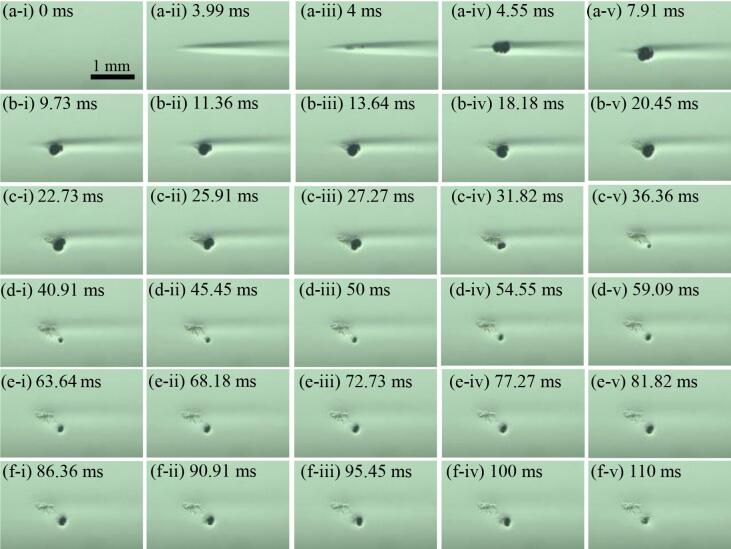

Additional high speed camera experiments were carried out in order to investigate the effects of changes in pressure-modulated shockwave histotripsy pulse length on the lifetime of a boiling bubble induced at the HIFU focus. A single 50 or 100 ms-long 2 MHz pressure-modulated shockwave histotripsy pulse was employed with *P*_1,_*_+_* = 86.1 MPa; *P*_1,−_ = –14.6 MPa and *P*_2,_*_+_* = 29.9 MPa; *P*_2,−_ = –9.6 MPa. Fig. 8 shows a series of high-speed images obtained over 50 ms. Boiling bubbles generated (indicated by the red arrow in Fig. 8a-iv) within the localised heated zone start to merge after *t* = 4 ms (Fig. 8b-i to d-i) followed by the formation of a larger bubble with a diameter of 304 μm at *t* = 7.27 ms (indicated by the red arrow in Fig. 8d-i). This enlarged bubble as a result of the coalescence process is then pushed away from the HIFU focus by the HIFU radiation force [19] (Fig. 8d-ii to f-i). This translational bubble movement can also contribute to damaging the tissue phantom. When the bubble is pushed outside the localised shockwave heated zone (i.e., axial length of 2.5 mm and lateral width of 0.19 mm), the bubble size gets smaller and smaller over time (Fig. 8f-ii to h-iv), but still persisting within the full-width half-maximum HIFU focal volume until the HIFU is switched off at *t* = 50 ms (Fig. 8h-iv). Similar results described above are observed under a 100 ms-long pressure-modulated shockwave histotripsy excitation, which are shown in Supplementary Fig. 1. During the experiments, no evidence of thermal damage, which would manifest itself as an opaque lesion in the tissue phantom [2], was present.

#### 3.2.2 3.5 MHz and 5 MHz

**Fig. 9:**
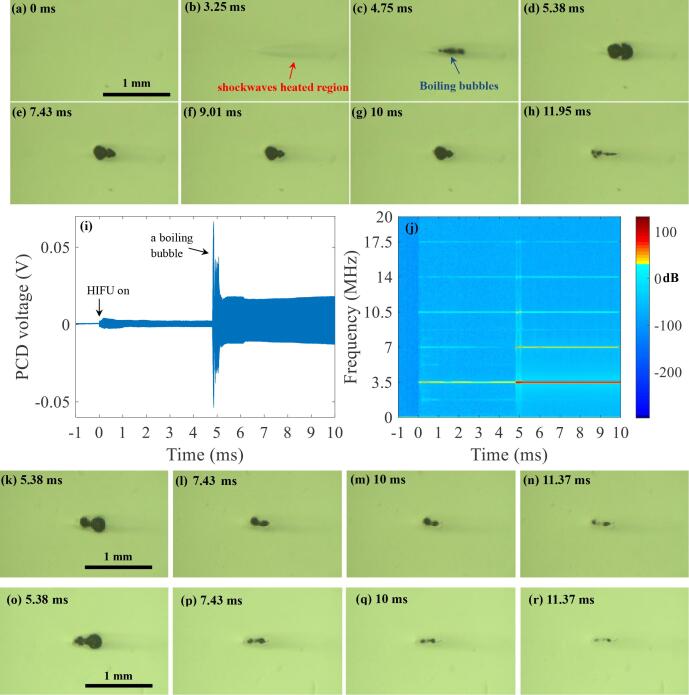
High speed images (a-h and k-r) taken during a single 10 ms-long 3.5 MHz pressure-modulated shockwave histotripsy pulse. (a to h) with *P*_1,_*_+_* of 72.4 MPa, *P*_1,−_ of –13.8 MPa and *P*_2,_*_+_* of 32.1 MPa, *P*_2,−_ of –9.6 MPa. (k to n) with *P*_2,_*_+_* of 17.6 MPa, *P*_2,−_ of –7.7 MPa. (o to r) with *P*_2,_*_+_* of 8.8 MPa; *P*_2,−_ of –5.4 MPa. Images (h), (n) and (r) were obtained 1.95, 1.37 and 1.37 ms after the end of the exposure respectively. (i) PCD voltage versus time plot in correspondence with (a to g). (j) the corresponding spectrogram of (i). The 3.5 MHz HIFU beam propagates from left to right. A movie showing the bubble dynamics captured during the course of the 10 ms-long 3.5 MHz pressure-modulated shockwave histotripsy exposure with *P*_1_,_+_ = 72.4 MPa; *P*_1_,_−_ = –13.8 MPa, and *P*_2_,_+_ = 32.1 MPa; *P*_2_,_−_ = –9.6 MPa is available in Supplementary Video 3.

**Fig. 10:**
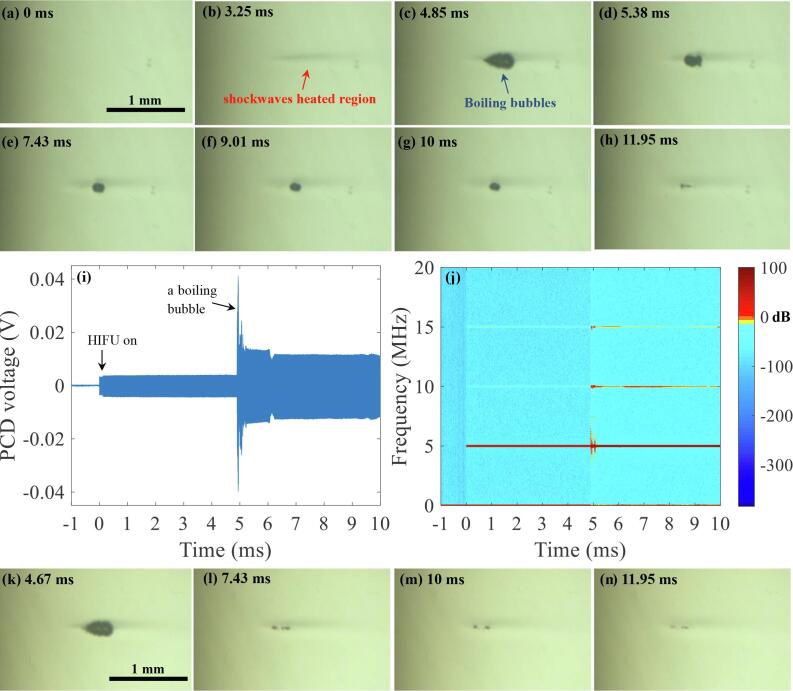
High speed images (a-h and k-n) taken during a single 10 ms-long 5.0 MHz pressure-modulated shockwave histotripsy pulse. (a to h) with *P*_1,_*_+_* of 69.2 MPa, *P*_1,−_ of –12.5 MPa and *P*_2,_*_+_* of 29.2 MPa, *P*_2,−_ of –8.6 MPa. (k to n) with *P*_1,_*_+_* of 69.2 MPa, *P*_1,−_ of –12.5 MPa and *P*_2,_*_+_* of 13.9 MPa, *P*_2,−_ of –6.4 MPa. Images (h) and (n) were obtained 1.95 ms after the end of the exposure. (i) PCD voltage versus time plot in correspondence with (a to g). (j) the corresponding spectrogram of (i). The 5.0 MHz HIFU beam propagates from left to right. A movie showing the bubble dynamics captured during the course of the 10 ms-long 5.0 MHz pressure-modulated shockwave histotripsy exposure with *P*_1_,_+_ of 69.2 MPa and *P*_1_,_−_ of –12.5 MPa, and *P*_2_,_+_ of 29.2 and *P*_2_,_−_ of –8.6 MPa is available in Supplementary Video 4.

The feasibility of the proposed pressure-modulated shockwave histotripsy was also investigated with the 3.5 and 5.0 MHz HIFU transducers, which are shown in Fig. 9, Fig. 10. This was conducted to examine whether the proposed histotripsy can also be performed with higher frequencies. The exposure conditions listed in Table 2 were employed here. In the experiments, *P*_+,1_ of 72.4 MPa and *P_−_*_,1_ of −13.8 MPa were kept constant during the first 5 ms of a 10 ms long-pressure modulated 3.5 MHz HIFU pulse, whilst those during the subsequent 5 ms of the exposure (i.e. a time period between 5 ms and 10 ms) were varied as: (a) *P*_+,2_ = 32.1 MPa and *P_−_*_,2_ = −9.6 MPa, (b) *P*_+,2_ = 17.6 MPa and *P_−_*_,2_ = −7.7 MPa, or (c) *P*_+,2_ = 8.8 MPa and *P_−_*_,2_ = −5.4 MPa. With the 5 MHz HIFU transducer, *P*_+,1_ of 69.2 MPa and *P_−_*_,1_ of −12.5 MPa were kept constant during the first 5 ms of a 10 ms long-pressure modulated 5.0 MHz HIFU pulse, and *P*_+,2_ and *P_−_*_,2_ for the subsequent 5 ms of the exposure (i.e., a time period between 5 ms and 10 ms) were as follows: (a) 29.2 MPa and −8.6 MPa, or (b) 13.9 MPa and −6.4 MPa.

Similar to the results obtained with the 2 MHz transducer, localised shockwave heating can be seen at the HIFU focus (Figs. 9b and 10b) followed by the production of boiling bubbles. During the experiments, the time to boiling for the single 3.5 MHz HIFU pulse with *P_+_*_,1_ of 72.4 MPa and *P_−_*_,1_ of –13.8 MPa in the liver tissue phantom was 4.38 ms ± 0.34 ms (mean ± standard deviation SD with *n* = 21) whilst that for the 5.0 MHz with *P_+_*_,1_ = 69.2 MPa and *P_−_*_,1_ = –12.5 MPa was 4.72 ms ± 0.24 ms (*n* = 23). In both the excitation cases (3.5 MHz and 5 MHz), no significant cavitation clouds are detected after *t* = 5 ms with the high speed camera and the PCD systems used in the present study (Fig. 9, Fig. 10). No significant broadband emissions appear whilst stronger and higher order multiple harmonics of the fundamental frequency occur in the spectrogram along with the formation of a boiling bubble. With the 3.5 MHz HIFU transducer, for instance, higher order multiple harmonic components with larger amplitudes can be seen at 3.5, 7, 10.5, 14 and 17.5 MHz (Fig. 9j), whereas these are observed at 5, 10 and 15 MHz with the 5 MHz HIFU source (Fig. 10j) in the corresponding spectrograms.

In the experiments performed with the 3.5 MHz HIFU transducer, residual mechanical damage with a size of 0.41 ± 0.07 mm (length, *n* = 6) × 0.15 ± 0.02 mm (width, *n* = 6) appears at *P*_2,+_ of 32.1 MPa and *P*_2,−_ of −9.6 MPa, whereas a smaller lesion of a 0.30 ± 0.01 mm (length, *n* = 3) × 0.08 ± 0.007 mm (width, *n* = 3) is attained with *P*_2,+_ of 8.8 MPa and *P*_2,−_ of −5.4 MPa. Similarly, a larger lesion forms at the HIFU focus with higher *P_2_*_,+_ and *P_2_*_,−_ under the 5 MHz excitation case. (i.e., With *P*_2,+_ of 29.2 MPa, *P*_2,−_ of −8.6 MPa: a size of 0.34 ± 0.02 mm (length, *n* = 5) × 0.06 ± 0.009 mm (width, *n* = 5). With *P*_2,+_ of 13.9 MPa, *P*_2,−_ of −6.4 MPa: a size of 0.28 ± 0.03 mm (length, *n* = 3) × 0.05 ± 0.013 mm (width, *n* = 3)). When comparing to the results obtained with the 2 MHz HIFU transducer, it can be clearly noticed that a smaller lesion can be produced with higher frequencies (Table 3).

## 4 Discussion

In the present study, a new histotripsy approach termed pressure-modulated shockwave histotripsy was proposed and demonstrated with high speed camera experiments. This method involves controlling the size and lifetime of boiling vapour bubbles through modulating peak pressure magnitudes (Fig. 5, Fig. 7, Fig. 9, Fig. 10) and a pulse length (Fig. 8, Supplementary Fig. 1) without inducing the shock scattering effect. A similar concept has been proposed in [28] where a short, high-intensity sequence of pulses is applied to initiate tissue erosion *via* inertial cavitation clouds and subsequent lower intensity pulses are employed to sustain the process. The main idea behind this method [28] is, however, to generate cavitation nuclei using high intensity ultrasound pulses which can provide cavitation seeds for the latter lower intensity pulses to sustain cavitation and erosion (i.e., focused on increasing the probability of tissue erosion), which is different to the concept proposed in the present study.

Maxwell et al [17] experimentally observed that the scattering of incident shockwaves from the surface of a single cavitating bubble in a tissue gel phantom can produce a large peak negative pressure field in front of the bubble, leading to additional bubble nucleation sites for an inertial cavitation cloud. This is the main mechanism involved in shock scattering histotripsy. Recent numerical and experimental results reported in [14], [19] clearly demonstrate the appearance of the constructive interference of the backscattered shockwave by a boiling bubble with the incoming incident shockwaves during boiling histotripsy exposure. It was found that this interaction can eventually induce a greater peak negative pressure field between the HIFU transducer and the bubble compared to that without the bubble at the HIFU focus [14], eventually leading to the formation of an additional inertial cavitation cluster [10], [15], [16], [19]. When the negative pressure magnitude of a backscattered acoustic field exceeds the cavitation intrinsic threshold [17], [18], a violent cavitation cluster is almost certain to form, which is known as the shock scattering effect. With the boiling histotripsy exposure condition used in the present study, the shock scattering effect was observed in Fig. 4(h) (indicated by the arrow) with the occurrence of strong broadband emissions in the spectrogram (Fig. 4p). On the contrary, no significant cavitation clouds and broadband emissions were detected after the formation of a boiling bubble under the proposed pressure-modulated shockwave histotripsy exposure (Fig. 5, Fig. 9, Fig. 10). This is most probably because the peak negative pressure magnitude of the backscattered acoustic field by the boiling bubble is below the cavitation cloud intrinsic threshold (Fig. 6b and c). Shear stresses induced around a boiling bubble can therefore lead to a localised mechanical fractionation at the HIFU focus, resulting in the formation of an oval shaped lesion with a length of 0.61 mm and a width of 0.14 mm (Fig. 5z-ii) as opposed to a tadpole shape (length: 2.72 mm, width: 0.25 mm, Fig. 4n), which is a typical lesion shape produced by boiling histotripsy.

During the proposed histotripsy exposure, bubble coalescence phenomenon, which is the process by which two or more bubbles merge during contact to form a single bubble, was clearly observed (Fig. 5, Fig. 7, Fig. 8). This coalescence is most likely to be due to the primary and secondary Bjerknes forces (i.e., translational forces on individual bubbles in an acoustic field), which are mainly dependent upon the bubble size relative to the sonication frequency [29]. According to Bjerknes’ theory [30], [31], bubbles which are smaller than the size that is resonant with the acoustic field (i.e. resonance bubble size at a given ultrasonic frequency) are migrated up a pressure gradient and are collected at the pressure antinodes, whereas bubbles of larger than the resonant size travel down a pressure gradient and aggregate at the pressure nodes [32], [33], [34]. This is known as the primary Bjerknes force. The secondary Bjerknes force appears between neighbouring bubbles in the same acoustic field. An attractive force is introduced between bubbles that are of similar size (i.e., their phase difference of oscillation is less than π/2) whilst a repulsive force forms between bubbles that are very different in size (i.e., their phase difference of oscillation is greater than π/2) [32], [33], [34], [35]. With the insonation frequency (2 MHz) used in the present study, a resonant bubble diameter is around 3 μm [10], which is much smaller than the size of the boiling bubbles observed in the present study (i.e., ranging from 149 to 217 μm, Fig. 5t). In the context of Bjerknes’ theory described above, it can be suggested that the primary Bjerknes force likely leads to the migration of boiling bubbles up the pressure gradient toward the antinode whilst the secondary Bjerknes force facilitates the attraction of these bubbles during the course of the proposed histotripsy exposure. At excessive ultrasonic intensity, it has been reported that bubble clustering is predominantly due to the secondary Bjerknes force. This secondary Bjerknes force increases with ultrasonic intensity more significantly than the primary Bjerknes force [29], [36]. The high speed images depicted in Fig. 5, Fig. 7 indicate this positive relationship of the degree of bubble coalescence with the peak pressure magnitudes where maximal coalescence was observed at the highest pressure amplitudes tested in the present study (i.e., *P*_2,+_ of 29.9 and *P*_2,−_ of –9.6 MPa).

In this study, the peak pressure amplitudes (*P*_2,+_ and *P*_2,−_) and the pulse length were varied in order to investigate the effects of changes in exposure conditions on final lesion production in the proposed pressure-modulated shockwave histotripsy. It was observed that the lesion size gradually increased with *P*_2,+_ and *P*_2,−_ (Figs. 5z-ii, 7, 8, 9, 10 and Table 3). Since there were no significant cavitation clouds appeared during the proposed histotripsy exposure, the extent of mechanical damage induced should mainly be attributable to that of a boiling bubble. Indeed, the size of a boiling bubble measured at the end of the pressure-modulated shockwave histotripsy exposure increased with increasing *P*_2,+_ and *P*_2,−_: 74.7 μm (Fig. 7b), 99.2 μm (Fig. 7a) and 298 μm (Fig. 5z-i) at (a) *P*_2,+_ = 5 MPa; *P*_2,−_ = –3.7 MPa, (b) *P*_2,+_ = 13.2 MPa; *P*_2,−_ = –6.8 MPa, and (c) *P*_2,+_ = 29.9 MPa; *P*_2,−_ = –9.6 MPa, respectively. This increase in the bubble size is most probably be due to the positive relationships between (a) the degree of bubble coalescence and the peak positive pressure amplitude (Fig. 5, Fig. 7) [29], [36], and (b) the size of a bubble with the peak negative pressure [16]. The feasibility of using the proposed histotripsy in controlling the lifetime of a boiling bubble at the HIFU focus was also demonstrated in the present study (Fig. 8, Supplementary Fig. 1). Under the exposure conditions used, a boiling bubble that initially formed by shock wave heating persisted and maintained within the HIFU focal zone until the end of the exposure (i.e., over 10, 50, or 100 ms), suggesting the possibility of varying the degree of mechanical damage through changing a pressure-modulated shockwave histotripsy pulse length (i.e., the longer the bubble lifetime, the higher the degree of damage produced, and vice versa). This warrants further investigation.

On the basis of the results presented in this study, it can be suggested that the proposed histotripsy could potentially be employed to induce a spatially localised tissue fractionation with a desired degree of mechanical damage through controlling the extent and lifetime of a boiling bubble without inducing the shock scattering effect. Instead of applying the proposed histotripsy method, one would suggest using an intense boiling histotripsy pulse that is just long enough to generate boiling bubbles (e.g., Fig. 4g). In boiling histotripsy, however, the explosion of a boiling bubble and its further interaction with shocks can cause mechanical tissue fractionation. Previous studies [2], [6] have reported that a boiling histotripsy pulse length needs to be longer than the time-to-boil (i.e., longer than the time to form initial boiling bubbles) so that the extent of mechanical damage induced by an exploding boiling bubble (*via* rectified bubble growth [10], [12], [13]) and its further interaction with incoming incident shockwaves (shock scattering effect [14], [15], [19], diffraction of the incoming HIFU waves [15], atomisation [37]) becomes larger than that of potential thermal injury by shockwave heating. In the present study, it can be observed that the width of a localised shockwave heated region (Fig. 5e) is larger than the size of an initial boiling bubble generated at the HIFU focus (Fig. 5g). In boiling histotripsy, inertial cavitation clouds form along with the explosive growth of a boiling bubble [2], [10], [14], [15], [16], [19], whereas this cavitation cluster disappears whilst keeping the boiling bubble at the HIFU focus with the proposed pressure-modulated shockwave histotripsy (Fig. 5, Fig. 7, Fig. 8). For effectively treating solid tumours, the proposed histotripsy can be used alongside with boiling histotripsy. A large solid tumour could, for instance, be initially treated with boiling histotripsy and the proposed pressure-modulated shockwave histotripsy could subsequently be employed to destroy the remnant tumour adjacent to normal tissue or major blood vessels.

## 5 Conclusions

In this study, pressure-modulated shockwave histotripsy was proposed and demonstrated. The concept of the proposed method is to control the extent and lifetime of boiling vapour bubbles through modulating peak pressure magnitudes and a pulse length without inducing the shock scattering effect. The high speed camera and the PCD experimental results presented clearly demonstrate that no cavitation clouds which typically appear between a boiling bubble and the HIFU transducer in boiling histotripsy occur under the proposed histotripsy exposure (i.e., no shock scattering effect appears). This is likely because that the peak negative pressure magnitude of a backscattered acoustic field by a boiling bubble is being kept below the cavitation cloud intrinsic threshold. A spatially localised mechanical damage, therefore, predominantly occurs as a result of the shear stresses produced around a boiling bubble at the HIFU focus, resulting in the creation of a smaller lesion in the shape of an oval-like than a typical tadpole shaped lesion produced by boiling histotripsy. Further investigations of the production of a lesion in *in vivo* tissue using the proposed histotripsy approach are necessary.

## CRediT authorship contribution statement

**Ki Joo Pahk:** Conceptualization, Data curation, Formal analysis, Funding acquisition, Investigation, Methodology, Supervision, Writing – original draft.

## Declaration of Competing Interest

The author declares that he has no known competing financial interests or personal relationships that could have appeared to influence the work reported in this paper.

