# Supplementary material for: Control of the dynamics of a boiling vapour bubble using pressure-modulated high intensity focused ultrasound without the shock scattering effect: A first proof-of-concept study
Source: Ultrason Sonochem. 2021 Jul 31;77:105699. doi: 10.1016/j.ultsonch.2021.105699 (PMC8358471; doi:10.1016/j.ultsonch.2021.105699)
Supplement: Supplementary data 5 [file mmc5.docx]

**Supplementary Figure and Tables**

Supplementary Figure 1. A sequence of images obtained during a 100 ms-long pressure-modulated shockwave histotripsy excitation with *P*_1,_*_+_* of 86.1 MPa; *P*_1,_*_-_* of –14.6 MPa and *P*_2,_*_+_* of 29.9 MPa; *P*_2,_*_-_* of –9.6 MPa. The peak pressures were changed at *t* = 4 ms. The 2 MHz HIFU beam propagates from left to right.

Supplementary Table 1. Composition of 200 mL tissue phantom with 7% concentration of BSA. BSA = bovine serum albumen. TRIS = trisaminomethane. APS = ammonium persulfate. TEMED = tetramethylethylenediamine.

| **Components** | **Quantity** | **Percent (%)** |
| --- | --- | --- |
| Degassed and de-ionised water | 143.22 mL | 71.61 |
| BSA | 14 g | 7 |
| 1 M TRIS | 20 mL | 10 |
| 40% Acrylamide | 35 mL | 17.5 |
| 10% APS | 1.68 mL | 0.84 |
| TEMED | 0.1 mL | 0.05 |

Supplementary Table 2. Acoustic and thermal properties of the liver tissue phantom used in the simulations performed in the present study. These values were obtained from [3].

| **Properties** | **Value** |
| --- | --- |
| Sound speed | 1544 m s^-1^ |
| Mass density | 1044 kg m^-3^ |
| Absorption coefficient at 1 MHz | 15 dB m^-1^ MHz^-1^ |
| Coefficient of nonlinearity | 4.0 |
| Specific heat capacity per unit volume | 5.3 × 10^6^ J m^-3^ ^o^C^-1^ |
| Thermal diffusivity | 1.3 × 10^-7^ J m^-2^ s^-1^ |
| Ambient temperature | 20 ^o^C |

**Supplementary Videos**

**Supplementary Video 1.** A movie showing the bubble dynamics captured over the 10 ms-long 2 MHz pressure-modulated shockwave histotripsy pulse with *P*_1_,_+_ of 89.1 and *P*_1_,_-_ of –14.6 MPa, and *P*_2_,_+_ of 29.9 and *P*_2_,_-_ of –9.6 MPa.

**Supplementary Video 2.** A movie showing the bubble dynamics captured during the course of the 50 ms-long 2 MHz pressure-modulated shockwave histotripsy exposure with *P*_1_,_+_ of 89.1 and *P*_1_,_-_ of –14.6 MPa, and *P*_2_,_+_ of 29.9 and *P*_2_,_-_ of –9.6 MPa.

**Supplementary Video 3.** A movie showing the bubble dynamics captured over the 10 ms-long 3.5 MHz pressure-modulated shockwave histotripsy pulse with *P*_1_,_+_ of 72.4 and *P*_1_,_-_ of –13.8 MPa, and *P*_2_,_+_ of 32.1 and *P*_2_,_-_ of –9.6 MPa.

**Supplementary Video 4.** A movie showing the bubble dynamics captured during the course of the 10 ms-long 5.0 MHz pressure-modulated shockwave histotripsy exposure with *P*_1_,_+_ of 69.2 and *P*_1_,_-_ of –12.5 MPa, and *P*_2_,_+_ of 29.2 and *P*_2_,_-_ of –8.6 MPa.
